# Uncovering the Latent Components of Physical Performance in Professional Soccer: Evidence from the Turkish First Division

**DOI:** 10.3390/jfmk10040434

**Published:** 2025-11-07

**Authors:** Spyridon Plakias, Dimitris Tsaopoulos, Themistoklis Tsatalas, Giannis Giakas

**Affiliations:** 1 Department of Physical Education and Sport Science, University of Thessaly, 42150 Trikala, Greece; ttsatalas@uth.gr (T.T.); ggiakas@gmail.com (G.G.); 2 Center for Research and Technology Hellas, 60361 Volos, Greece; dtsaop@gmail.com

**Keywords:** football, match demands, external load, factor analysis, playing positions

## Abstract

**Background:** Physical performance in soccer is usually described through isolated indicators such as total distance or sprint frequency, which may overlook the broader structure of match demands. **Purpose:** This study aimed to identify the latent components of physical performance in professional soccer and to examine how they vary across playing positions. **Methods:** External load data were collected from 446 outfield players competing in the Turkish first division during the 2021–2022 season, using optical tracking technology. Distances covered at different speed thresholds and maximal speed were analyzed through principal component analysis. Factor scores were compared across positions using non-parametric tests. **Results:** Three components of physical performance emerged: (1) moderate-intensity running (2–5.5 m/s, inverse to low-speed activity), (2) high-intensity running (>5.5 m/s), and (3) sprint capacity (maximal speed). Central midfielders recorded the highest values in moderate-intensity running, wingers and wing backs excelled in high-intensity running, while sprint capacity was most strongly associated with wingers. **Conclusions:** The findings provide a more integrated understanding of soccer’s physical demands, moving beyond single indicators to reveal broader performance dimensions. This framework can support coaches, analysts, and scouts in player profiling, training design, and rehabilitation planning, while emphasizing the need for position-specific physical preparation.

## 1. Introduction

Soccer performance is influenced by a complex interplay of tactical, technical, psychological, and physical factors [1,2]. The physical demands of modern professional soccer involve greater work intensity and a more frequent competition schedule, requiring players to exert more effort than in past decades [3,4]. Furthermore, while the tactical, technical, and psychological demands are essential for determining match outcomes, the physical aspects not only contribute to performance but are also directly linked to player health, injury prevention, and support rehabilitation after injuries [5,6,7]. For these reasons, the physical demands of the game warrant particular attention from practitioners, and consequently, academic interest in the physical performance of male professional soccer players during competition has grown considerably in recent decades [8].

In recent years, considerable research has focused on the quantification of players’ external load during competitive matches through advanced tracking technologies [2,9,10]. GPS (Global Positioning System) and optical tracking technologies can provide large amounts of data, offering numerous promising research opportunities and addressing the long-standing issue of small sample sizes, which are more susceptible to bias [6,11]. Typical indicators include total distance covered, distance covered at different speed thresholds, the number of accelerations and decelerations, the number of high-speed runs, and the maximal speed [6,10,12]. Such metrics have been widely used to describe the physical demands of soccer players, but also to make comparisons between playing positions [12,13].

It has been widely accepted since the first time-motion analysis studies that there are significant differences in competitive physical activity profiles depending on the position, which are linked to the tactical demands specific to each role [8]. As shown in the recent review by Sarmento et al. [12] central and wide midfielders cover the greatest total distances during matches, averaging approximately 11,012 m and 10,894 m, respectively, with full backs also recording high values (10,457 m). In contrast, forwards (10,068 m) and central defenders (9598 m) cover considerably less ground. Regarding high-speed running, wide midfielders outperform all other positions, completing substantially more meters at high intensity (+106 m compared to full backs and +191 m compared to central midfielders). Central defenders and forwards show the lowest high-speed running values, while full-backs occupy an intermediate position, exceeding central defenders and forwards. Finally, in sprinting at maximum speed, wide midfielders again register the highest distances (330 m), followed by forwards (280 m) and full-backs (272 m). Central defenders record the lowest sprinting output (180 m), with central midfielders (224 m) also surpassing them.

However, all these studies, included in a review by Sarmento et al. [12], have examined physical indicators in isolation, which may lead to fragmented conclusions. To date, no efforts have been made to identify the underlying components of physical performance in soccer through data-driven approaches such as factor analysis, although this is a common practice when analyzing technical–tactical variables [14,15]. A better understanding of how these variables group together into broader performance dimensions could provide deeper insights into physical match demands and contribute to more precise performance profiling across playing positions. This would greatly assist team coaches, analysts, and scouts in selecting the right players for the roster, as well as in determining the starting eleven.

Addressing this gap, the present study aimed to identify the latent components of physical performance in professional soccer players using match-derived external load data, obtained with the Instatscout optical tracking method. Furthermore, positional differences across the extracted components were examined to provide a deeper understanding of how different playing roles are characterized by distinct performance dimensions. We hypothesized that (a) a small number of meaningful components would emerge from the data, and (b) these components would differ significantly across playing positions. By doing so, this study seeks to advance knowledge on the structure of physical performance in soccer, thereby offering practical applications for performance analysis, training design, and injury prevention.

## 2. Materials and Methods

### 2.1. Sample

The sample was drawn from 238 matches of the Turkish first division’s 2021–2022 season (all matches of the competition up to the 24th round, except for two matches for which data were missing). The initial dataset included 7262 observations (one observation for each player who participated in each match).

All observations (*n* = 1813) in which a player’s participation was less than 45 min were removed, leaving 5449 valid observations. Subsequently, mean values for each variable were calculated per player. For all variables (except maximal speed), normalization was performed with respect to playing time so that each player’s values referred to 90 min of play. This was carried out using the formula: Value = mean Variable × 90/mean time.

In this way, the overall dataset was constructed, comprising N = 485 players. Of these, 39 goalkeepers were excluded due to the specific nature of their position, resulting in the final dataset of 446 players. Goalkeepers are often excluded from research analyses, likely due to the unique characteristics of their role, which involves lower physical, physiological, and technical demands compared to outfield players [12].

### 2.2. Variables

**Figure 1:**
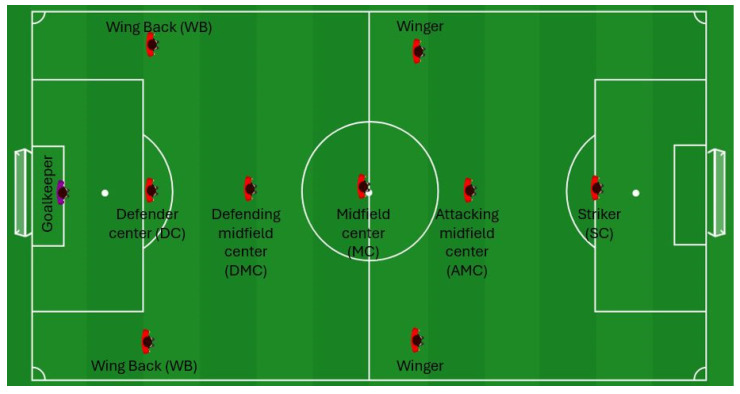
Schematic representation of the players’ primary positions on the pitch as categorized by Transfermarkt.

The dataset included the following variables: playing time, total distance, distance covered at speeds up to 2 m/s, distance covered at 2–4 m/s, distance covered at 4–5.5 m/s, distance covered at 5.5–7 m/s, distance covered at over 7 m/s, and maximal speed. The thresholds defining the distance covered in different intensity zones have also been applied in previous research [16,17,18]. Information regarding each player’s primary position was obtained from Transfermarkt (https://www.transfermarkt.com/ (accessed on 10 June 2025)). This website categorizes players into the following positions: goalkeeper, defender center (DC), wing back (WB), defending midfield center (DMC), midfield center (MC), attacking midfield center (AMC), winger, and striker (SC). Figure 1 provides a schematic representation of the players’ positions on the pitch. Data from Transfermarkt are considered reliable and have been widely used in numerous studies [19,20,21].

### 2.3. Procedure Ethics

The dataset was obtained through the optical tracking system provided by InStat (https://football.instatscout.com/ (accessed on 18 September 2022)). Notably, this system is FIFA-licensed and has demonstrated high levels of both absolute and relative reliability. A comprehensive report on its reliability is available on FIFA’s official website [22]. Furthermore, InStat’s tracking technology was employed as the official electronic performance and tracking system of the Turkish league during the 2021–2022 season.

Written consent was obtained from the company InStat Ltd. (Instat Limited Roselawn House, University Business Complex National Technology Park Castletroy Co., Limerick Ireland) on 8 November 2022, permitting the use of the data for research and publication purposes. Ethical approval for the study was subsequently granted by the Ethics Committee of the University of Thessaly on 12 October 2022 (approval code: 1973).

### 2.4. Statistical Analysis

Our initial dataset was imported into SPSS (version 29.0; IBM Corporation, Armonk, NY, USA), where the values were transformed into z-values. Using the z-transformed variables, a factor analysis–PCA was performed to identify the components of the physical performance of the players. Sampling adequacy and the suitability of the data for factor analysis were first evaluated using the Kaiser–Meyer–Olkin (KMO) measure and Bartlett’s test of sphericity. The variable total distance was excluded from the factor analysis because its inclusion resulted in a KMO value below the acceptable threshold of 0.5 [23]. The extraction of factors was based primarily on Cattell’s scree plot criterion (factors retained before the point of the sharpest inflection, where the curve tends to become parallel with the *x*-axis), while eigenvalues were also taken into account [24,25,26]. After all, as noted by Iantovics et al. [27], the combined use of the Kaiser Criterion and Cattell’s Scree test is often recommended to support a more accurate decision regarding the number of factors to be retained. For the rotation method, Varimax was applied, as the Component Correlation Matrix indicated low correlations between the three factors (all <0.10), suggesting that the extracted dimensions were largely independent. Factor loadings were displayed in a matrix sorted by size, with coefficients below |0.60| suppressed to enhance interpretability. In addition, factor scores for each player were computed and saved as new variables using the regression method. These scores were subsequently used in further analyses.

To compare the factor scores of physical performance’s components across playing positions, Kruskal–Wallis tests were conducted (one for each extracted factor). The Kruskal–Wallis test was chosen instead of the parametric equivalent (one-way ANOVA) because the Shapiro–Wilk tests indicated that the variables were not normally distributed across all groups (positions). When significant differences were detected, post hoc pairwise comparisons were performed using the Mann–Whitney U test, with *p*-values adjusted using the Bonferroni correction. The effect size r was calculated according to the formula r = z/√N, where z is the standardized test statistic and N is the total sample size (i.e., the sum of the two groups being compared). For interpretation, the following thresholds were applied: r = 0.1 (small effect), r = 0.3 (medium effect), and r = 0.5 (large effect) [28,29,30,31]. All statistical analyses were performed using SPSS, with the level of significance set at *p* < 0.05. The data for all original variables (playing time, total distance, distance covered at speeds up to 2 m/s, distance covered at 2–4 m/s, distance covered at 4–5.5 m/s, distance covered at 5.5–7 m/s, distance covered at over 7 m/s, and maximal speed) are presented as means and standard deviations. This approach is standard in performance analysis research and allows for a clear representation of central tendency and variability.

## 3. Results

### 3.1. Descriptive Statistics

Table 1 presents the descriptive statistics (mean and standard deviation) for the entire sample of players as well as for each position separately. It is worth noting that the values are normalized to 90 min of play and do not reflect a regular match including stoppage time.

### 3.2. Factor Analysis-PCA

**Figure 2:**
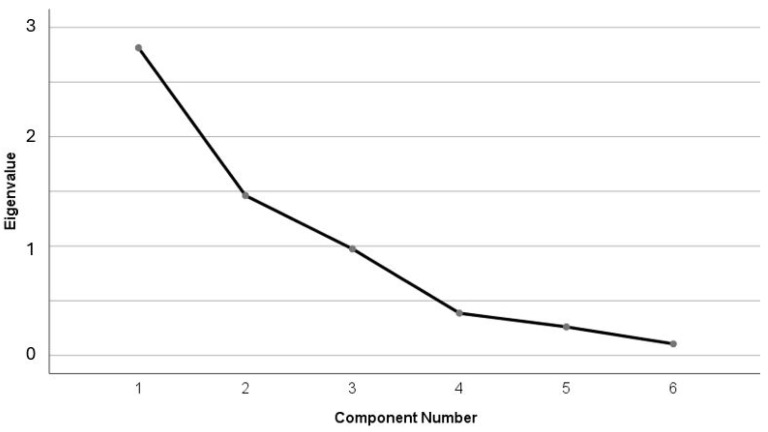
Scree plot of eigenvalues, showing a clear elbow at the fourth component, which supports the retention of a three-factor solution.

The suitability of the dataset for factor analysis was confirmed by Bartlett’s test of sphericity (χ^2^(15) = 1400.09, *p* < 0.001), while the KMO value was 0.616, indicating a moderate level of sampling adequacy. PCA with Varimax rotation was performed. Factor extraction was primarily based on Cattell’s scree plot (Figure 2), with eigenvalues also considered as supportive evidence.

Three components were retained, explaining 87.49% of the total variance (Factor 1 = 46.90%, Factor 2 = 24.36%, Factor 3 = 16.23%) (Table 2). Although the eigenvalue of the third factor was slightly below the Kaiser criterion threshold (0.974 < 1.0), it was nevertheless retained because (a) the scree plot clearly indicated a three-factor solution, (b) previous methodological recommendations have emphasized that rigid application of the eigenvalue-greater-than-one rule is questionable, since very small differences (e.g., 1.01 vs. 0.99) that may result from sampling error can lead to acceptance or rejection without substantive justification [26], and (c) the factor explained a substantial proportion of the variance (16.23%).

The rotated component matrix (Varimax rotation) showed a clear three-factor structure (Table 3). Factor 1 was defined by distances covered at 4–5.5 m/s (0.913) and 2–4 m/s (0.909), with a negative loading from distances covered up to 2 m/s (−0.858). Factor 2 was represented by high-intensity running, specifically distance covered over 7 m/s (0.923) and distance covered at 5.5–7 m/s (0.768). Factor 3 corresponded exclusively to maximal speed (0.998).

Overall, the PCA yielded a clear three-factor structure representing:

◦ Moderate-intensity running (distances at velocity between 2 m/s and 5.5 m/s, inverse to low-speed activity);
◦ High-intensity running (distances at velocity higher than 5.5 m/s);
◦ Sprint capacity (maximal speed).

### 3.3. Kruskal–Wallis and Mann–Whitney Tests

For the three factors that emerged, Kruskal–Wallis tests were conducted to examine whether there were differences across the various playing positions. In all three cases, the results were statistically significant (*p* < 0.001, df = 6). For the three Kruskal–Wallis tests, the H values were as follows: (a) for moderate-intensity running, H = 177.028; (b) for high-intensity running, H = 207.028; (c) for sprint capacity, H = 132.288.

**Figure 3:**
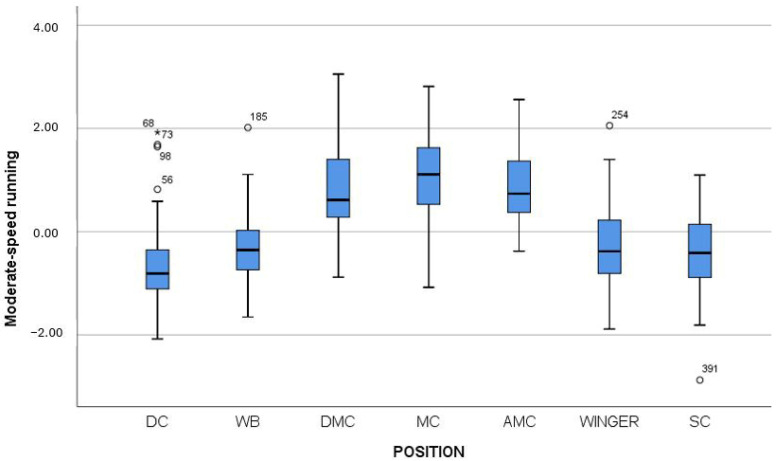
Box plot of the factor Moderate-speed running distance across player positions. Note: DC = Defender Center; WB = Wing Back; DMC = Defending Midfield Center; MC = Midfield Center; AMC = Attacking Midfield Center; Winger = Wide Midfielder; SC = Striker.

**Figure 4:**
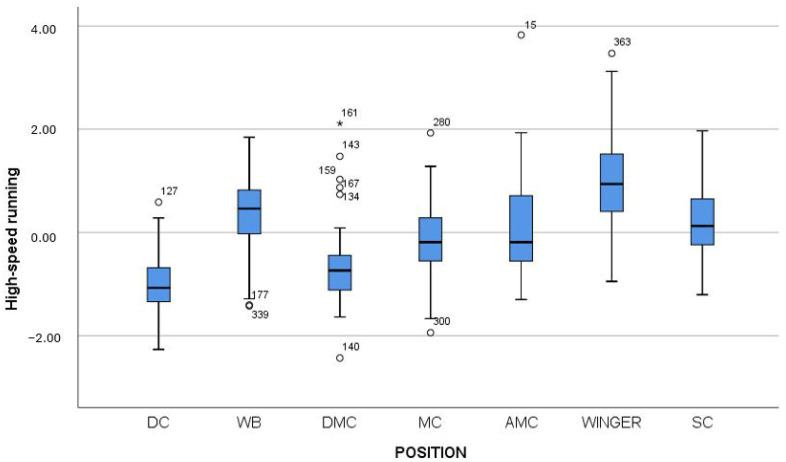
Box plot of the factor High-speed running distance across player positions. Note: DC = Defender Center; WB = Wing Back; DMC = Defending Midfield Center; MC = Midfield Center; AMC = Attacking Midfield Center; Winger = Wide Midfielder; SC = Striker.

**Figure 5:**
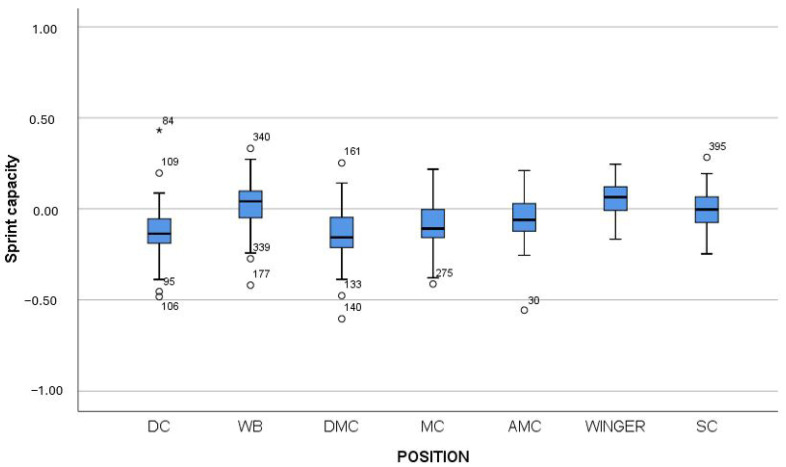
Box plot of the factor Sprint capacity across player positions. Note: DC = Defender Center; WB = Wing Back; DMC = Defending Midfield Center; MC = Midfield Center; AMC = Attacking Midfield Center; Winger = Wide Midfielder; SC = Striker.

Therefore, Mann–Whitney tests were subsequently performed to determine between which positions these differences occurred. Table 4, Table 5 and Table 6 present the results of all pairwise comparisons, while Figure 3, Figure 4 and Figure 5 provide a graphical representation of the results using box plots.

From Table 4 and Figure 3, it emerges that DMC, MC, and AMC players performed statistically significantly higher than the other positions on the factor Moderate-intensity running distance, with large effect sizes (r > 0.5). Additionally, wingers outperformed DCs, although the effect size was small to moderate (r = 0.244).

**Table 4:** Pairwise comparisons (Mann–Whitney U tests with Bonferroni adjustment) identifying between-position differences for the factor Moderate-intensity running.

Sample 1-Sample 2	Test Statistic	Std. Error	Std. Test Statistic	Sig.	Adj. Sig.	N	r
AMC-MC	13.895	26.785	0.519	0.604	1.000	96	0.053
DC-AMC	−209.767	24.710	−8.489	<0.001	<0.001	129	0.747
DC-DMC	−188.880	24.286	−7.777	<0.001	<0.001	131	0.679
DC-MC	−223.661	21.819	−10.251	<0.001	<0.001	147	0.845
DC-SC	−48.558	21.591	−2.249	0.025	0.515	149	0.184
DC-WB	−59.147	19.495	−3.034	0.002	0.051	175	0.229
DC-WINGER	−63.113	20.152	−3.132	0.002	0.036	165	0.244
DMC-AMC	−20.886	28.830	−0.724	0.469	1.000	80	0.081
DMC-MC	−34.781	26.395	−1.318	0.188	1.000	98	0.133
SC-AMC	161.209	26.600	6.060	<0.001	<0.001	98	0.612
SC-DMC	140.323	26.207	5.354	<0.001	<0.001	100	0.535
SC-MC	175.104	23.938	7.315	<0.001	<0.001	116	0.679
SC-WB	10.589	21.841	0.485	0.628	1.000	144	0.040
SC-WINGER	14.556	22.430	0.649	0.516	1.000	134	0.056
WB-AMC	−150.620	24.929	−6.042	<0.001	<0.001	124	0.543
WB-DMC	−129.733	24.508	−5.293	<0.001	<0.001	126	0.472
WB-MC	−164.514	22.066	−7.455	<0.001	<0.001	142	0.626
WB-WINGER	−3.966	20.420	−0.194	0.846	1.000	160	0.015
WINGER-AMC	146.653	25.446	5.763	<0.001	<0.001	114	0.540
WINGER-DMC	125.767	25.034	5.024	<0.001	<0.001	116	0.466
WINGER-MC	160.548	22.649	7.089	<0.001	<0.001	132	0.617

Note: DC = Defender Center; WB = Wing Back; DMC = Defending Midfield Center; MC = Midfield Center; AMC = Attacking Midfield Center; Winger = Wide Midfielder; SC = Striker.

From Table 5 and Figure 4, it can be observed that wingers performed statistically significantly higher than all other positions on the factor High-intensity running distance. They were followed by wing backs, who outperformed all other positions except strikers. Strikers showed significantly higher values than DCs, DMCs, and MCs, while AMCs outperformed DCs and DMCs. Finally, DCs displayed significantly lower values than all other positions, except DMCs.

**Table 5:** Pairwise comparisons (Mann–Whitney U tests with Bonferroni adjustment) identifying between-position differences for the factor High-intensity running.

Sample 1-Sample 2	Test Statistic	Std. Error	Std. Test Statistic	Sig.	Adj. Sig.	N	r
AMC-SC	−24.599	26.600	−0.925	0.355	1.000	98	0.093
AMC-WB	53.977	24.929	2.165	0.030	0.638	124	0.194
AMC-WINGER	−106.998	25.446	−4.205	<0.001	0.001	114	0.394
DC-AMC	−147.015	24.710	−5.950	<0.001	<0.001	129	0.524
DC-DMC	−52.831	24.286	−2.175	0.030	0.622	131	0.190
DC-MC	−123.611	21.819	−5.665	<0.001	<0.001	147	0.467
DC-SC	−171.615	21.591	−7.948	<0.001	<0.001	149	0.651
DC-WB	−200.992	19.495	−10.310	<0.001	<0.001	175	0.779
DC-WINGER	−254.013	20.152	−12.605	<0.001	<0.001	165	0.981
DMC-AMC	−94.184	28.830	−3.267	0.001	0.023	80	0.365
DMC-MC	−70.780	26.395	−2.682	0.007	0.154	98	0.271
DMC-SC	−118.784	26.207	−4.533	<0.001	<0.001	100	0.453
DMC-WB	148.161	24.508	6.045	<0.001	<0.001	126	0.539
DMC-WINGER	−201.182	25.034	−8.036	<0.001	<0.001	116	0.746
MC-AMC	−23.405	26.785	−0.874	0.382	1.000	96	0.089
MC-SC	−48.004	23.938	−2.005	0.045	0.944	116	0.186
MC-WB	77.382	22.066	3.507	<0.001	0.010	142	0.294
MC-WINGER	−130.403	22.649	−5.758	<0.001	<0.001	132	0.501
SC-WB	29.377	21.841	1.345	0.179	1.000	144	0.112
SC-WINGER	82.399	22.430	3.674	<0.001	0.005	134	0.317
WB-WINGER	−53.021	20.420	−2.597	0.009	0.198	160	0.205

Note: DC = Defender Center; WB = Wing Back; DMC = Defending Midfield Center; MC = Midfield Center; AMC = Attacking Midfield Center; Winger = Wide Midfielder; SC = Striker.

From Table 6 and Figure 5, it can be observed that wingers demonstrated statistically significantly higher values in Sprint capacity compared to almost all other positions (AMC, DMC, MC, SC, and DC). WBs also outperformed DCs, DMCs, AMCs, and MCs, while strikers showed significantly higher values than DMCs and MCs. Finally, AMCs recorded significantly lower values than WBs and wingers, whereas DMCs exhibited the lowest values overall, being significantly outperformed by WBs, wingers, MCs, and SCs. DCs also showed relatively low values, falling statistically significantly behind WBs, wingers, and SCs.

**Table 6:** Pairwise comparisons (Mann–Whitney U tests with Bonferroni adjustment) identifying between-position differences for the factor Sprint capacity.

Sample 1-Sample 2	Test Statistic	Std. Error	Std. Test Statistic	Sig.	Adj. Sig.	N	r
AMC-SC	−45.342	26.600	−1.705	0.088	1.000	98	0.172
AMC-WB	77.963	24.929	3.127	0.002	0.037	124	0.281
AMC-WINGER	−110.370	25.446	−4.337	<0.001	<0.001	114	0.406
DC-AMC	−68.001	24.710	−2.752	0.006	0.124	129	0.242
DC-MC	−30.271	21.819	−1.387	0.165	1.000	147	0.114
DC-SC	−113.343	21.591	−5.250	<0.001	<0.001	149	0.430
DC-WB	−145.964	19.495	−7.487	<0.001	<0.001	175	0.566
DC-WINGER	−178.371	20.152	−8.851	<0.001	<0.001	165	0.689
DMC-AMC	−75.736	28.830	−2.620	0.009	0.181	80	0.293
DMC-DC	7.735	24.286	0.319	0.750	1.000	131	0.028
DMC-MC	−38.006	26.395	−1.440	0.150	1.000	98	0.145
DMC-SC	−121.079	26.207	−4.620	<0.001	<0.001	100	0.462
DMC-WB	153.699	24.508	6.271	<0.001	<0.001	126	0.559
DMC-WINGER	−186.106	25.034	−7.434	<0.001	<0.001	116	0.690
MC-AMC	−37.730	26.785	−1.409	0.159	1.000	96	0.144
MC-SC	−83.073	23.938	−3.470	0.001	0.011	116	0.322
MC-WB	115.693	22.066	5.243	<0.001	<0.001	142	0.440
MC-WINGER	−148.100	22.649	−6.539	<0.001	<0.001	132	0.569
SC-WB	32.621	21.841	1.494	0.135	1.000	144	0.125
SC-WINGER	65.082	22.430	2.904	0.004	0.079	134	0.251
WB-WINGER	−32.407	20.420	−1.587	0.113	1.000	160	0.125

Note: DC = Defender Center; WB = Wing Back; DMC = Defending Midfield Center; MC = Midfield Center; AMC = Attacking Midfield Center; Winger = Wide Midfielder; SC = Striker.

## 4. Discussion

The aim of this study was to identify the latent components of physical performance in professional soccer using match-derived external load data and to examine positional differences across these components. Three dimensions of physical performance (moderate-intensity running, high-intensity running, sprint capacity) were identified with significant differences across playing positions, confirming our initial hypotheses. More specifically, midfielders (DMC, MC, AMC) recorded the highest values in moderate-intensity runs, while the highest values of high-intensity running were identified in wingers and wing backs, and sprint capacity was associated mostly with the wingers. The novelty of these findings comes from the examination of the latent structure of physical performance during match play, rather than the isolated analysis of a commonly used set of external load indicators. Identification of these components and the variability in them between the various positions contributes to a more integrative understanding of the physical demands of soccer for player profiling, position-specific training, and injury prevention strategies.

Although no previous research has identified the components of physical performance as such, when considering the variables that load on each factor, our findings are consistent with earlier studies. In particular, we found that wide players (wingers and WBs) reached higher maximal speeds compared to players in other positions, a result also reported in previous research [32,33]. This can be explained tactically by the fact that wide players frequently operate in situations where they can exploit available space. Unlike central players, who often act in congested zones with limited room and heavy defensive pressure, wide players more often have the opportunity to run through open corridors (flanks) with greater distance ahead of them [12]. Especially during transitions, wingers and WBs are required to cover long stretches of the pitch at maximal speed, either to support attacking actions or to recover defensively [34,35]. This reflects the demands of modern soccer, where coaches expect WBs not to remain solely in defense but to advance and provide attacking width when the team is in possession. Conversely, wingers are often required to track back and support defensively, either by doubling up against the opposing winger or by covering the weak side when the ball is played to the opposite flank and the WB tucks inside to maintain compact distances within the defensive line.

The ability of wide players to exploit large spaces and corridors explains why these players also cover the greatest distances at high-speed intensities (>5.5 m/s). This finding is consistent with previous research. For example, Ingebrigtsen et al. [36] reported that players in lateral positions covered significantly greater high-speed distances compared to central players, both in each half separately and across the full match duration. Moreover, this pattern appears to be consistent across different populations, as has also been confirmed in studies on female players [37]. In addition, numerous studies have demonstrated that midfielders (both central and wide) are the players who accumulate the greatest total distance during matches [38,39,40]. Given that wide midfielders also cover larger distances at high speeds, it is reasonable that, within the factor Moderate-speed running distance, the different types of central midfielders (DMC, MC, AMC) emerged with the highest values. This outcome reflects their pivotal role in match play, as central midfielders typically occupy positions of high centrality in network analyses, constantly linking defensive and offensive actions and thereby requiring sustained moderate-intensity running across all phases of the game [35,41].

Furthermore, these positional differences are also influenced by the distinct physiological adaptations and typical training emphases associated with each playing role. Wide players are frequently involved in training and match scenarios that require repeated high-speed runs and sprints along wide corridors, which enhance their ability to sustain high-intensity efforts. In contrast, central midfielders regularly engage in activities that involve continuous movement and mixed-intensity efforts, reflecting their role as links between defense and attack. Over time, these positional demands contribute to differences in aerobic capacity, running economy, and anaerobic speed reserve, which are reflected in the observed physical performance profiles [42,43,44,45,46].

A major strength of the present study is its data-driven approach to identifying and interpreting the latent components of match-derived physical performance. Contrary to previous studies on this subject that used and interpreted single indicators (e.g., total distance, number of sprints, distances covered at various intensity levels), the present study provides a framework for combining match-derived variables into meaningful dimensions. It is worth noting that Oliva-Lozano et al. [47] also identified three components but focused on constructing a composite index and did not examine positional differences for the individual components. Furthermore, we analyzed variability across seven distinct outfield playing positions, whereas their positional classification was limited to only three broad categories (defenders, midfielders, forwards), which likely contributed to the small effect sizes they observed. This contribution fills a critical gap in the international literature, offering a novel conceptualization of soccer’s physical demands. Beyond its academic value, the findings carry important practical implications. Scouts, coaches, and performance analysts can use these components to better understand the positional requirements of modern soccer, thereby improving talent identification and roster composition. Moreover, the results provide benchmarks for rehabilitation and return-to-play processes, helping practitioners determine the physical capacities players must regain after injury according to their playing position.

Despite these strengths, the present study also has certain limitations that should be acknowledged. First, the dataset was derived from a single league (Turkish first division), which may limit the generalizability of the findings to other competitions with different tactical and physical demands. Second, only external load indicators were analyzed, while no internal load or physiological measures (e.g., heart rate, lactate, RPE) were included, which could have provided a more comprehensive picture of players’ physical performance [48,49,50]. Third, the data for the players’ position (e.g., WB, DC, SC, etc.) refer to the main position in which they play; however, this does not mean that their coaches did not use them in a different position in some games (or part of them). Fourth, contextual variables such as tactical formation, playing style, match status, opponent quality, and environmental conditions were not considered, although they are known to influence physical outputs [18,51,52,53,54]. Finally, the cross-sectional nature of the analysis does not account for within-player variability across a season or potential longitudinal adaptations. Future research should therefore aim to replicate these findings in other leagues and competitive levels, integrate more external load variables (e.g., accelerations and decelerations) and internal load measures, and adopt longitudinal designs to capture changes in physical performance components over time.

## 5. Conclusions

This study revealed three key components of physical performance in professional soccer (moderate-intensity running, high-intensity running, and sprint capacity), each showing clear differences across playing positions. Central midfielders were characterized by sustained moderate-intensity activity, wingers and wing backs stood out for their high-intensity running, and sprint capacity was most evident among wingers. By moving beyond single variables and instead identifying broader dimensions of performance, this work offers a more integrated understanding of the physical demands of modern soccer.

From a practical perspective, these findings provide useful information for coaches, analysts, and scouts when designing training programs, profiling players, or planning rehabilitation targets after injury. At the same time, the study highlights the importance of tailoring physical preparation to the unique requirements of each position. Future research should build on this framework by including data from different leagues and competitive levels, integrating internal load measures, and tracking changes over time. In doing so, we can move closer to a holistic view of physical performance in soccer that connects research insights with applied practice on the field.

## Figures and Tables

**Table 1:** Mean and standard deviation (SD) for the total sample as well as for each position separately.

Position	Cases	Statistic	Total Distance (m)	Distance on Speed up to 2 m/s (m)	Distance on Speed 2–4 m/s (m)	Distance on Speed 4–5.5 m/s (m)	Distance on Speed 5.5–7 m/s (m)	Distance on Speed over 7 m/s (m)	Maximal Speed (m/s)
Total	446	Mean	9958.36	3447.59	3901.58	1715.47	748.35	147.20	8.45
		SD	761.97	237.76	462.56	389.52	176.65	77.12	0.38
DC	90	Mean	9219.38	3522.49	3688.47	1373.39	543.91	92.19	8.33
		SD	567.69	194.62	354.58	265.33	117.62	37.96	0.35
WB	85	Mean	9835.51	3493.46	3786.29	1608.71	758.66	190.72	8.66
		SD	538.76	198.00	357.17	256.47	117.12	61.23	0.32
DMC	41	Mean	10,326.39	3278.57	4243.71	1989.20	723.14	93.38	8.17
		SD	664.68	222.53	426.71	354.66	168.30	61.68	0.42
MC	57	Mean	10,666.06	3253.14	4324.69	2141.66	835.70	112.85	8.23
		SD	539.85	214.45	353.43	341.38	143.16	62.37	0.36
AMC	39	Mean	10,625.42	3316.68	4258.44	2057.69	862.88	131.63	8.33
		SD	514.39	186.43	355.52	279.15	159.07	76.60	0.38
Winger	75	Mean	10,029.33	3527.68	3739.27	1690.69	857.22	216.59	8.68
		SD	667.84	231.75	436.98	301.52	150.08	74.98	0.26
SC	59	Mean	9791.99	3557.30	3716.69	1594.41	764.41	161.10	8.53
		SD	648.02	218.52	433.80	265.11	127.77	57.71	0.30

Note: DC = Defender Center; WB = Wing Back; DMC = Defending Midfield Center; MC = Midfield Center; AMC = Attacking Midfield Center; Winger = Wide Midfielder; SC = Striker.

**Table 2:** Total variance explained by the extracted components, including initial eigenvalues, extraction sums of squared loadings, and rotation sums of squared loadings.

Component	Initial Eigenvalues			Extraction Sums of Squared Loadings			Rotation Sums of Squared Loadings		
	Total	% of Variance	Cumulative %	Total	% of Variance	Cumulative %	Total	% of Variance	Cumulative %
1	2.814	46.895	46.895	2.814	46.895	46.895	2.747	45.791	45.791
2	1.462	24.363	71.258	1.462	24.363	71.258	1.499	24.978	70.769
3	0.974	16.229	87.487	0.974	16.229	87.487	1.003	16.718	87.487
4	0.386	6.43	93.917						
5	0.26	4.334	98.252						
6	0.105	1.748	100						

**Table 3:** Rotated component matrix with Varimax rotation, showing factor loadings for the three extracted components.

Initial Variables	Components		
	1	2	3
Zscore (Distance on speed 4–5.5 m/s)	0.913		
Zscore (Distance on speed 2–4 m/s)	0.909		
Zscore (Distance on speed up to 2 m/s)	−0.858		
Zscore (Distance on speed over 7 m/s)		0.923	
Zscore (Distance on speed 5.5–7 m/s)		0.768	
Zscore (Maximal speed)			0.998

## Data Availability

The datasets generated and analyzed during the current study consist of player performance data and are therefore subject to privacy and ethical restrictions. For this reason, they are not publicly available. However, the data is available from the corresponding author upon reasonable request.

## Abbreviations

The following abbreviations are used in this manuscript:

GPS	Global Positioning System
DC	Defender Center
WB	Wing Back
DMC	Defending Midfield Center
MC	Midfield Center
SC	Striker
